# Controlled variations in stimulus similarity during learning determine visual discrimination capacity in freely moving mice

**DOI:** 10.1038/srep01048

**Published:** 2013-01-10

**Authors:** Mario Treviño, Tatiana Oviedo, Patrick Jendritza, Shi-Bin Li, Georg Köhr, Rodrigo J. De Marco

**Affiliations:** 1 Department of Molecular Neurobiology Max-Planck-Institute for Medical Research, Heidelberg, Germany; 2 Developmental Genetics of Nervous System Max-Planck-Institute for Medical Research, Heidelberg, Germany

## Abstract

The mouse is receiving growing interest as a model organism for studying visual perception. However, little is known about how discrimination and learning interact to produce visual conditioned responses. Here, we adapted a two-alternative forced-choice visual discrimination task for mice and examined how training with equiprobable stimuli of varying similarity influenced conditioned response and discrimination performance as a function of learning. Our results indicate that the slope of the gradients in similarity during training determined the learning rate, the maximum performance and the threshold for successful discrimination. Moreover, the learning process obeyed an inverse relationship between discrimination performance and discriminative resolution, implying that sensitivity within a similarity range cannot be improved without sacrificing performance in another. Our study demonstrates how the interplay between discrimination and learning controls visual discrimination capacity and introduces a new training protocol with quantitative measures to study perceptual learning and visually-guided behavior in freely moving mice.

Psychophysics and recordings of neuronal activity have long been used to study vision in monkeys, cats and humans. More recently, it has been shown that rodent visual circuits bear many similarities to those in these species. For instance, neurons in the mouse primary visual cortex have highly tuned receptive fields^1^,^2^, and mice can discriminate simple^3^,^4^ and complex^5^ shapes. Thus, the mouse is currently emerging as an important and practical model system for studying the neuronal circuitry underlying visual discrimination, perceptual learning and decision-making^6^.

In the mouse, however, visual discrimination can only be studied through learning, and learning, in turn, improves discrimination performance^3^,^4^. Little consideration has been given to the question of how the interplay between visual discrimination and learning influences the development of discrimination capacity and conditioned response in freely moving mice. Experience-dependent improvements in discrimination performance have been reported in most, if not all, auditory^7^, visual^8^,^9^ and olfactory^10^ tasks. When a conditioned stimulus (CS^+^) is held constant, the learning rate increases with the discriminative value of the CS^+^, thereby making stimulus discriminability a powerful determinant of how perceptual learning transfers between analogous visual stimuli^8^,^11^. However, the relationship between varying stimulus discriminability and learning remains poorly understood. This is highly relevant because open environments vary continuously and allow locomotion, which modifies the structure of sensory arrays^12^, and little is known about how learning and discrimination deal with such variability. We reasoned that, if the discriminability of a reinforced stimulus varies continuously during learning, then the sign and slope of such stimulus variations should determine the learning rate and shift the discrimination threshold.

To study the interplay between visual discrimination and learning, we adapted a two-alternative forced-choice visual discrimination task^3^,^4^ and examined how positive, negative and oscillating gradients of stimulus similarity correlated with conditioned response and discrimination performance as a function of learning. During training, we presented the mice with a fixed reinforced image (*i.e.* conditioned stimulus, CS^+^) and multiple non-reinforced images (CS^−^) with different degrees of structural similarity to the CS^+^, measured by using parametric descriptions derived from image quality metrics. This allowed us to arrange equiprobable CS^−^ stimuli into different training configurations of variable similarity. Introducing novel measures that allow the detection of successful discrimination of complex images, we found that the difficulty of comparable training conditions shaped the development of a well-defined visual conditioned response in freely moving mice^3^,^4^. Our results reveal the rules that govern the interplay between discrimination and learning.

## Results

### Heterogeneous visual stimuli and discrimination task

**Figure 1:**
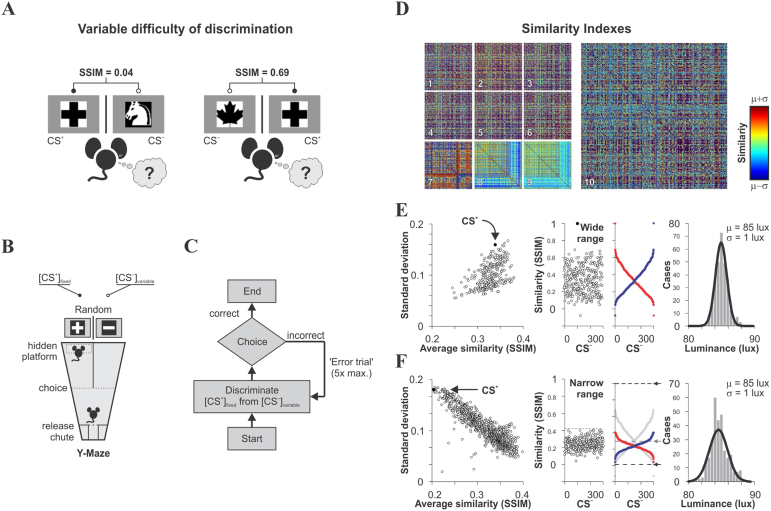
Visual discrimination task and training paradigm with heterogeneous stimulus similarity. (A) Sample CS^+^ with exchangeable CS^−^ stimuli for discriminative trials. The difficulty in discrimination depends on the degree of structural similarity (SSIM) between stimuli, indicated on the top. (B) Scheme of the visual discrimination swimming task: two monitors facing the ends of the arms of a Y-maze simultaneously display the positive (CS^+^, reinforced) and a negative (CS^−^, non-reinforced) stimuli (100% contrast). A submerged transparent platform below the CS^+^ serves as the unconditioned stimulus (US). The position of both the platform and CS^+^ in either arm varies pseudo-randomly over consecutive trials. During training, mice are released into the pool from a release chute and they learn to swim towards the CS^+^ (correct choice) in order to reach the platform and escape from the water. (C) Flowchart of a ‘training unit’ where the mice are presented with a given pair of CS^+^/CS^−^, a presentation that can be repeated up to 5 times if the mouse makes incorrect choices. (D) Color matrices showing similarity comparisons across all combinations of image pairs. Comparisons for the first set of stimuli are on the left (1–9): SSIM, 2D-xcorr, SNR, Weighted SNR, Peak SNR, MSE, NQM, VIF, and VIFP. The SSIM-matrix for 1112 hybrid images is displayed on the right. Color-bar on the right; red indicates greater and blue lower similarity between images. (E–F) Standard deviation against average SSIM for each reference image with respect to the remaining stimuli, with the training CS^+^ depicted as a black dot. Note the well distributed similarities spanning into a ‘wide’ (E) or a ‘narrow’ range (F). CS^−^ stimuli can be sorted by increasing (blue dots) or decreasing (red dots) similarity relative to the CS^+^, used as the stimulus timeline for visual discrimination training. The wide and narrow SSIM training regimes only differ in the sorting of equiprobable stimuli. The luminance distributions on the right (Set_1_: μ ± σ: 85 ± 1 lux, *n* = 300; Set_2_: μ ± σ: 84 ± 1 lux, *n* = 300) confirm that the stimuli can be treated as equiluminant.

Learning is inferred from the relationships between the discriminative stimuli to be learned (input) and the ensuing learned response (output). In a simple two-alternative discrimination task (Fig. 1A, B), a subject has to make a decision between two hypotheses (one of which leads to reward) in the presence of some degree of uncertainty^13^,^14^. Decision confidence increases with the degree of discriminability of the conditioned stimulus (CS^+^;^10^,^15^,^16^,^17^,^18^,^19^). To investigate the effect of varying CS^+^ discriminability on visual discrimination performance and conditioned response, we generated a set of visual stimuli with different degrees of structural similarity among them. We created 300 black-and-white, equiluminant, low-pass filtered, heterogeneous images with different irregularities in shape (see examples in Fig. 1A; see also Methods). To estimate the relative structure of the images, we applied parametric descriptions from image quality metrics (see Methods), and made cross-comparisons between all combinations of image pairs (Fig. 1D). The structural similarity index (SSIM^20^), for example, compares local patterns of pixel intensities, already normalized for luminance and contrast. We then selected an image to be used as a CS^+^ (filled symbol in Fig. 1E), with similarities with respect to the remaining non-rewarded CS^−^ images ranging from −0.07 to 1 (average SSIM = 0.33 ± 0.01, *n* = 300; hereafter referred to as ‘wide’ similarity range). Moreover, to implement an additional training regime covering a narrower similarity range and smaller average similarity, we created 1112 hybrid images using linear combinations of the above stimuli, before selecting a second set of 300 new images with narrower range in similarity (0.04 ≤ SSIM ≤ 0.39) and smaller average similarity (average SSIM = 0.23 ± 0.01; Fig. 1F; hereafter referred to as ‘narrow’ similarity range). The same CS^+^ was used for the restrained ‘narrow’ and the unrestrained ‘wide’ training regimes.

### Learning with fixed CS^+^/CS^−^ similarity

**Figure 2:**
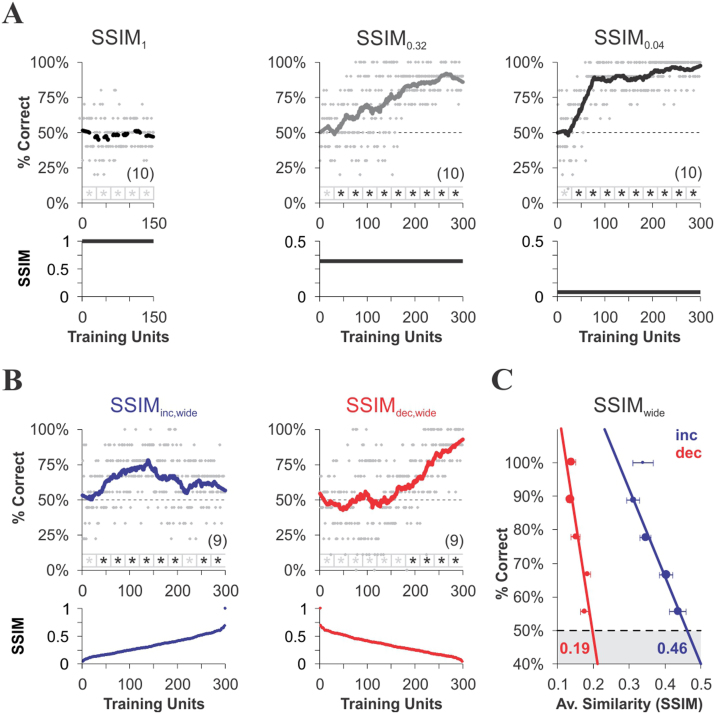
Learning with constant and varying CS^+^ discriminability. Acquisition curves (thick lines, approximated by a Savitzky-Golay filter) for mice trained with different temporal arrangements of CS^+^/CS^−^ similarity (SSIM). Thin dashed lines correspond to chance level at 50% correct choices. (A) Training with a constant SSIM of 1 (left, dashed), 0.32 (middle, gray) or 0.04 (right, black). (B) Training with heterogeneous CS^−^ stimuli, linearly sorted into increasing (blue) or decreasing (red) SSIM values over a ‘wide’ SSIM range. Asterisks below charts depict choice values above chance level (Wilcoxon test, *P < 0.05). The average performance at 150 ± 5 training units with identical average SSIM was higher for the increasing similarity group (SSIM_inc,wide_: 70% ± 5%, SSIM_dec,wide_: 51% ± 6%; one-way ANOVA, *F*_1,15_ = 4.13, *P* = 0.04). (C) Correct choice probability against average stimulus similarity for the corresponding trials from each group. The data can be approximated by weighted linear regressions (dot size proportional to number of cases per condition). Note how training influences the slope and intersection (with chance level) of the regression lines (thresholds depicted in bold letters). Number of mice per group in parentheses.

**Figure 3:**
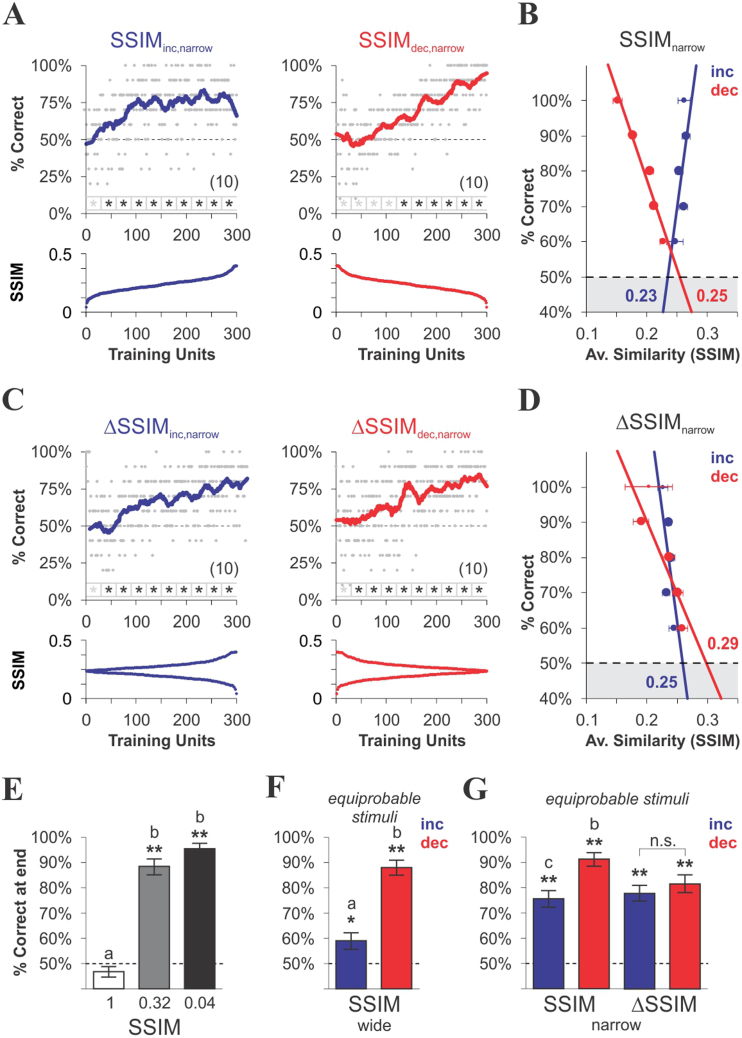
Learning with varying CS^+^ discriminability in a narrow range of CS^+^/CS^−^ similarity. Acquisition curves (thick lines, approximated by a Savitzky-Golay filter) for mice trained with different temporal arrangements of CS^+^/CS^−^ similarity (SSIM). Thin dashed lines correspond to chance level at 50% correct choices. (A) Training with equiprobable CS^−^ stimuli, linearly sorted into increasing (blue) or decreasing (red) SSIM values from a ‘narrow’ SSIM range. (C) Training with the same stimuli as in (A) arranged into increasing (blue) or decreasing (red) gradients of inter-training unit similarity (ΔSSIM); this oscillating arrangement maximizes similarity gradients, whereas linear sorting minimizes them. Asterisks below charts depict above chance choice values (Wilcoxon test, *P < 0.05). The average performance at 150 ± 5 training units with identical average SSIM was: SSIM_inc,narrow_: 69% ± 5%, SSIM_dec,narrow_: 61% ± 6%, ΔSSIM_inc,narrow_: 82% ± 3%, ΔSSIM_dec,narrow_: 65% ± 3%; one-way ANOVA, *F*_3,35_ = 9.44, *P* = 0.02. (B, D) Correct choice probability against average stimulus similarity for the corresponding trials from each group. Pooling the data from the 6 groups trained with varying similarity rendered a linear regression with a clear negative slope, an adjusted R^2^ = 0.99, and an overall similarity discrimination threshold of SSIM ≈ 0.33 (not illustrated), and shuffling the SSIM data by random permutations rendered infinite slopes (not illustrated). (E–G) Average performance of last 30 training units at the end of training. Asterisks indicate above chance choice values (Wilcoxon test, *P < 0.05, **P < 0.01) and lowercase letters depict the differences between groups (paired-comparisons, one-way ANOVA, followed by Bonferroni's post-tests, *P* < 0.05). Number of mice per group in parentheses.

Having defined these sets of equiluminant stimuli, we set out to study how mice learn to discriminate between CS^+^ and CS^−^ images offering different degrees of structural similarity over the course of acquisition. To achieve this, we adapted a two-alternative forced-choice visual discrimination task (Fig. 1A–C^3^,^4^). Initial quick improvements in task performance are considered evidence of “procedural” learning (*i.e.* task-specific response calibrations^7^), whereas subsequent and more gradual improvements are considered evidence of “perceptual” or “stimulus” learning^11^,^21^. To minimize the contribution of procedural learning on visual discrimination learning, we exposed the mice from each group to an initial one-week period of ‘pre-training’ (150 training units), which allowed them to become familiar with the swimming pool and the task, and learn to assign an initial CS_0_^+^ image with predictive value (Figs. S1–S3). All the groups performed similarly at the end of this first week (one-way ANOVA, *F*_8,78_ = 12.86, *P* = 0.11; see also Methods). During the second phase lasting two weeks (300 training units in Figs. 2 and 3; see also Figs. S1–S3), we trained the mice to discriminate between pairs of images presented over consecutive trials, using a fixed reinforced image (CS^+^ different to the CS_0_^+^ from phase 1; see Methods) and a non-reinforced image (CS^−^) from the above set of stimuli. Thus, CS^+^ discriminability was indirectly specified by exchanging CS^−^ stimuli during the second phase of training. In these conditions, the mice had to continuously pay attention to both the CS^+^ and CS^−^ images as stimuli configuration changed from trial to trial.

We began by evaluating correct choice probability as a function of training in three initial groups that were trained with fixed CS^+^/CS^−^ similarity (SSIM) of maximum (1, SSIM_1_), intermediate (0.32, SSIM_0.32_) and low (0.04, SSIM_0.04_) levels (Fig. 2A; see also Fig. S1). These groups allowed us to investigate how fixed but different levels of stimulus similarity led to specific learning rate and maximum discrimination performance^22^. As expected, the mice failed to discriminate between identical CS^+^ and CS^−^ images in SSIM_1_ (Fig. 2A, left, dashed line; Fig. 3E, white bar; Wilcoxon test, *P* = 0.17, *n* = 10). By contrast, training with a constant SSIM of either 0.32 (Fig. 2A, middle, grey) or 0.04 (Fig. 2A, right, black) yielded learning curves with above random choice level (SSIM_0.32_: 84.9% ± 1.8%, *n* = 10; SSIM_0.04_: 91.3% ± 1.2%, *n* = 10; Fig. 3E, grey and black bars, respectively; Wilcoxon test, *P* < 0.01). Despite the fact that these groups had different learning rates (SSIM_0.32_: 0.63%/trial, *n* = 10; SSIM_0.04_: 2.02%/trial, *n* = 10; Figs. 2A** and **S1), they both reached similar correct choice levels at the end of training (Fig. 3E; one-way ANOVA, *F*_2,29 _ = 123.5, *P* < 0.001, Bonferroni's post hoc test, *P* > 0.05). These results confirm that learning rate increases by lowering the CS^+^/CS^−^ similarity, in our specific experimental conditions. In addition, increments in choice performance were relatively slow and retained across daily sessions^23^.

### Learning with positive, negative and oscillating gradients of CS^+^/CS^−^ similarity

Sensory representations depend on the physical properties of the perceived stimuli, yet the build-up of discriminative information is sensitive to prior response and reward probabilities^13^. This prompted us to investigate how the mice learn to discriminate between pairs of images that offer varying degrees of structural similarity over the course of training. We hypothesized that the sign and slope of steady variations in SSIM (*i.e.* similarity) values, using a fixed set of visual stimuli should exert measurable effects on discrimination learning. To assess this, we compared data from mice trained with equiprobable CS^−^ stimuli, linearly sorted into increasing (SSIM_inc,wide_) or decreasing (SSIM_dec,wide_) SSIM values (*i.e.* sustained positive or negative gradients; Fig. 1E blue and red dots, respectively). Importantly, these two groups involved exactly the same training stimuli within the ‘wide’ CS^+^/CS^−^ similarity range (Figs. 2B and 3F; see also Fig. S2). Shortly after the beginning of training, we observed a growing correct choice level in the increasing similarity group (SSIM_inc,wide_), peaking (68.8% ± 3.3%, *n* = 9), and then slightly decreasing when the similarity between the stimuli became too high (Fig. 2B). By contrast, the decreasing similarity group (SSIM_dec,wide_) displayed a slower onset for correct choice (SSIM_inc,wide_: 55 trials *versus* SSIM_dec,wide_: 215 trials) and higher maximal performance (SSIM_dec,wide_: 81.6% ± 3.3%, *n* = 9; Fig. 2B, see also Fig. S2). To investigate the overall relationship between correct choice and stimulus similarity during discriminative trials, we calculated the average SSIM of all image pairs satisfying group choice probabilities given by [correct_mice/total_mice]. The resulting discrete performance values (y-axis) against the average SSIM (x-axis) for each training regime are plotted in Fig. 2C and were well described by weighted linear relationships. Remarkably, each training program was associated with a specific slope and discrimination threshold (*i.e.* the intersection of the linear regression with chance level), which was 0.46 for SSIM_inc,wide_ and 0.19 for SSIM_dec,wide_, revealing flexible visual performance.

Based on these results, we trained two additional groups of mice (SSIM_inc,narrow_ and SSIM_dec,narrow_; Fig. 3A, B, ‘narrow’ similarity range; see also Fig. S3A, B) in which similarity was kept below the discrimination threshold obtained with SSIM_inc,wide_ (*i.e.* SSIM = 0.46; Fig. 2C). This allowed us to re-examine the relationship between visual discrimination and learning in situations where CS^+^ discriminability remained uncompromised. In these ‘narrow’ conditions, correct choice level reached a plateau at maximal performance (76.4% ± 2.7%, *n* = 10) in the SSIM_inc,narrow _group (Fig. 3A) while the decreasing similarity group displayed a slower onset for correct choice (SSIM_inc,narrow_: 45 trials vs. SSIM_dec,narrow_: 120 trials) and higher peak performance (SSIM_dec,narrow_: 86.8% ± 2.6%, *n* = 10; Fig. 3A, see also Fig. S3A, B). Linear regressions for choice *versus* average similarity are shown in Fig. 3B, with thresholds of 0.23 for SSIM_inc,narrow_ and 0.25 for SSIM_dec,narrow_. These results confirm that training with decreasing similarity yielded a higher maximum performance than training with increasing similarity (Fig. 3F, ANOVA, *F*_3,37_ = 22.3, *P* < 0.001, Bonferroni's post hoc tests, *P* < 0.05 when comparing pairs among all groups), thereby influencing learning rate (SSIM_inc,wide_: 1.53%/trial; SSIM_dec,wide_: 3.28%/trial; SSIM_inc,narrow_: 1.01%/trial; SSIM_dec,narrow_: 0.73%/trial). Notably all four training regimes led to above random choice levels (Fig. 3F, Wilcoxon test, *P < 0.05, **P < 0.01), including SSIM_inc,wide_ with maximum SSIM values at the end of training (Fig. 2B, blue), indicating that the mice effectively learned to discriminate between very similar images. Interestingly, training with decreasing similarity led to similar performance levels for SSIM_dec,wide_ and SSIM_dec,narrow _at the end of training, despite the fact that discrimination was potentially compromised in SSIM_dec,wide_, due to the higher SSIM values in the wider range of similarity. The slower onset and higher learning rate in SSIM_dec,wide _might reflect some form of task-irrelevant visual perceptual learning^24^.

Finally, we assessed two additional groups of mice trained with increasing (ΔSSIM_inc,narrow_) or decreasing (ΔSSIM_dec,narrow_) inter-training unit gradients of CS^+^/CS^−^ similarity within the narrower similarity range. Using such oscillating gradients, we investigated how variations in discrimination difficulty imposed either at the beginning or at the end of training influenced learning. To our surprise, both ΔSSIM groups (Fig. 3C; see also Fig. S3C, D) displayed similar maximum performance (ΔSSIM_inc,narrow_: 71.9% ± 2.6%, *n* = 10; ΔSSIM_dec,narrow_: 75.9% ± 1.9%, *n* = 10) and similar learning rate (ΔSSIM_inc,narrow_: 1.20%/trials; ΔSSIM_dec,narrow_: 0.84%/trials). Thus, training with either increasing or decreasing inter-training unit similarity gradients led to comparable (Fig. 3G, t test, *P* = 0.44) above random choice level (Wilcoxon test, **P < 0.01). We conclude that the observed differences in conditioned response between the linearly increasing and decreasing similarity groups cannot be accounted for by disruptions in the normal course of acquisition, as seen from the fact that both groups subjected to inter-training unit similarity variations (ΔSSIM_dec,narrow_ and ΔSSIM_inc,narrow_) yielded similar learning curves.

Because maximum retention level depends on the number of training repetitions^22^, we compared the number of swimming trials required to solve the task (*i.e.* to complete phase 2 of training) for each experimental group. As expected, the mice trained with SSIM_0.04_ required the lowest number (SSIM_1_: 569 trials ± 8 trials, *n* = 10; SSIM_0.32_: 420 trials ± 7 trials, *n* = 10; SSIM_0.04_: 370 trials ± 6 trials, *n* = 10; one-way ANOVA, *F*_2,26 _ = 25.3, *P* < 0.001), whereas both the groups trained with wide (SSIM_inc,wide_: 470 trials ± 12 trials, *n* = 9; SSIM_dec,wide_: 491 trials ± 5 trials, *n* = 9; one-way ANOVA, *F*_1,15 _ = 3.6, *P* = 0.058) and narrow SSIM range (SSIM_inc,narrow_: 435 trials ± 11 trials, *n* = 10; SSIM_dec,narrow_: 448 trials ± 10 trials, *n* = 10; ΔSSIM_inc,narrow_: 447 trials ± 7 trials, *n* = 10; ΔSSIM_dec,narrow_: 459 trials ± 8 trials, *n* = 10; one-way ANOVA, *F*_3,35 _ = 5.2, *P* = 0.158) required similar amount of swimming trials to complete training. Therefore, the differences in discrimination performance (Fig. 3F–G) cannot be explained by differences in training trials. Additional comparisons and swimming efficiency as a function of training are provided in Fig. S4.

### Decay of retention during extinction

**Figure 4:**
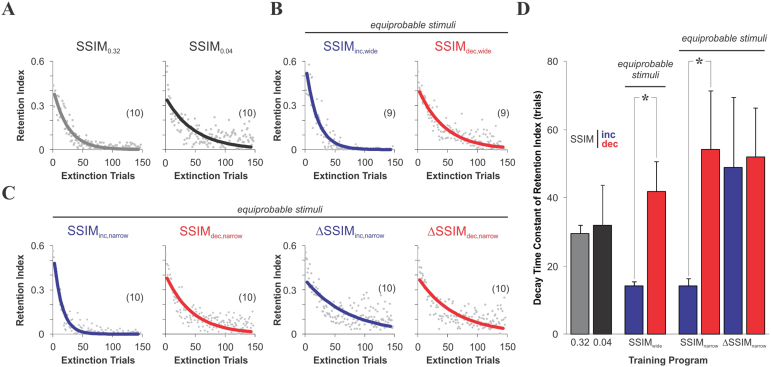
Decay of retention levels during extinction. Extinction tests were conducted with the same CS^+^ used during phase 2 of training and a fixed CS^−^ (common to all groups) with very low similarity against the CS^+^ (SSIM = 0.04) in the absence of reinforcement (*i.e.* the submerged platform was removed; see Methods). (A–C) Group average values of the retention index as a function of the extinction trials. The decay was approximated by a mono-exponential process (continuous lines). Number of mice per group in parentheses. (D) Mono-exponential curves were non-linear fit to the data from individual mice and their decay-times compared across groups. Note the apparently slower decay in the retention index obtained with SSIM_dec,narrow_ compared to SSIM = 0.04. Asterisks denote differences between groups (unpaired t test, *P < 0.05).

To further examine the impact of the different training regimes on discrimination learning, we conducted an ‘extinction’ test^22^,^25^ with a fixed low similarity to monitor the decline of the conditioned response in the absence of reinforcement. As expected, key features of the conditioned response (*v.gr.* choice, path-length and escape latency) decreased with the number of extinction trials (data not shown). To enhance the sensitivity of this test, we calculated a ‘retention index’ as the accumulated distance travelled in the CS^+^-arm divided by the total path length, whose slow decline improved the detection of group-differences (Fig. 4A–C). Interestingly, training with increasing similarity led to faster extinction decays than with decreasing similarity, both for the ‘wide’ and ‘narrow’ SSIM regimes (SSIM_inc,wide_: τ = 14.2 ± 1.5 training units; SSIM_dec,wide_: τ = 42.0 ± 8.8 training units; SSIM_inc,narrow_: τ = 14.3 ± 2.3 training units; SSIM_dec,narrow_: τ = 54.2 ± 17.4 training units; unpaired t test, *P* < 0.05; Fig. 4D). This difference was absent between the groups trained with either increasing or decreasing inter-training unit gradients in SSIM (ΔSSIM_inc,narrow_: τ = 52.2 ± 22.2 training units; ΔSSIM_dec,narrow_: τ = 55.6 ± 15.6 training units; unpaired t test, *P* = 0.89), and also between the groups trained with constant SSIM (SSIM_0.32_: τ = 29.5 ± 2.6 training units; SSIM_0.04_: τ = 31.9 ± 11.8 training units; unpaired t test, *P* = 0.66; Fig. 4B). Therefore, the sign of the similarity gradients determined the retention level not only during acquisition (Figs. 2**, **3 and S1–S4A), but also during extinction. However, although the extinction tests were conducted with a very low degree of CS^+^/CS^−^ similarity, common to all groups, it is still unclear how the different levels of performance at the end of training (Fig. 3E–G) relate to the subsequent decay during extinction (see also^26^).

### Conditioned response as a function of training with stimuli of varying similarity

**Figure 5:**
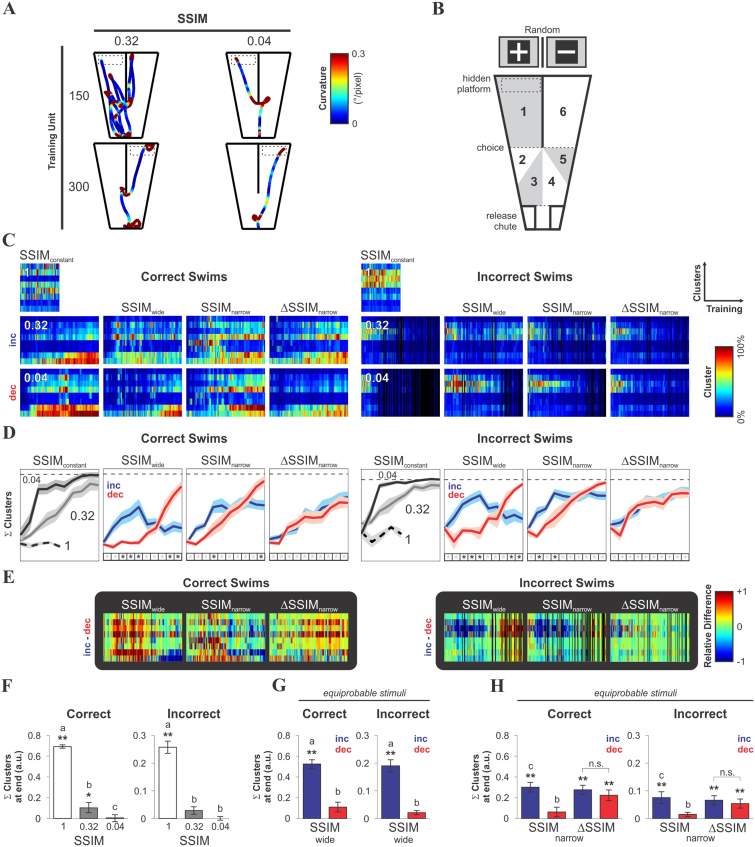
Conditioned response as a function of training with varying stimulus discriminability. (A) Sample trajectories at training units 150 and 300 (rows) of mice trained with a constant SSIM of either 0.32 (left) or 0.04 (right), with the local curvature represented in color (see Methods). (B) Scheme of the swimming pool, divided into 6 ‘regions of interest’, defined in terms of their locations relative to the CS^+^ image. Using data on the path length and cumulative curvature from each sub-region, pattern vectors for each swimming trial were calculated, pooled and fuzzy-clustered using principal component analysis (see also Fig. S5). Regions are symmetric and allow combined access to CS^+^ and CS^−^ (regions 3 and 4), visual access to CS^+^ or CS^−^ before making a choice (regions 2 or 5) or visual access to CS^+^ or CS^−^ after making a choice (regions 1 or 6). (C) Group average cluster maps from the cluster analysis of the mice's swimming trajectory as a function of their training; the data from correct (left) and incorrect (right) swims are shown separately. Color designates the distance to the center of mass for each of the 8 identified clusters, piled up as rows (color-bar on the right). (D) Average sum of all clusters per training unit (line plots; mean ± S.E.M) shows the evolution of conditioned responses as a function of training for correct and incorrect swims. Asterisks depict differences between groups (Two-way repeated measures ANOVA tests followed by Bonferroni's post hoc tests, *P < 0.05). (E) Point-to-point subtraction between the above cluster maps from (C) is shown on black background. Color-bar on the right. Black vertical lines in panels C and E arise from insufficient data to estimate the average clusters for the given training unit. (F–H) Group comparisons of conditioned responses at the end of training, as measured by the difference from SSIM_0.04_. Asterisks depict differences against the reference (Wilcoxon test, *P < 0.05, **P < 0.01); lowercase letters depict across-groups differences (one-way ANOVAs, followed by Bonferroni's post hoc tests, or Kruskal-Wallis tests, followed by Dunn's multiple comparison tests, *P* < 0.05; see also Methods).

In perceptual discrimination, the ability of sensory-motor circuits to integrate sensory evidence over time is thought to underlie the process of decision-making^13^,^27^,^28^. Movement enhances the capacity to collect discriminative information^29^ because the different viewpoints produced through locomotion transform the optic array, increasing the amount of sensory information^12^. With this in mind, we first compared the data from discrete choice measures with that of the continuous measures from the mice's swimming trajectories (Figs. S1–S3). Both path-length and escape latencies decreased as learning progressed, with their values tending towards asymptotic levels, likely minimizing the cost of adaptive behavior^13^. However, the probability of observing ‘efficient’ CS^+^-guided behavior was strongly influenced by the training regime, since increasing stimulus similarity produced higher error rates, path lengths and escape latencies, measures that depend on visual processing, stimulus discriminability and motor action^27^,^30^,^31^,^32^. To explore this further, we analyzed the swimming paths of the mice as a function of their training. Despite the evident complexity of the trajectories, we could detect substantial differences between the groups by simple visual inspection. For example, in Fig. 5A we show the swimming paths of two mice belonging to different training regimes (columns, SSIM_0.32_, and SSIM_0.04_) at two training stages (rows, training unit 150, 300; see also Fig. S5A). We hypothesized that paths of variable length and curvature would occur in conditions that range from ‘random’ to ‘optimal’ CS^+^-guided swimming. To test this possibility, we extracted specific topographic information from each swimming trial. First, we divided the swimming pool into six ‘regions of interest’ (1 to 6) that were defined in accordance to the CS^+^ position (Fig. 5B): regions 3 and 4 allowed visual access to both the CS^+^ and CS^−^ images, whereas regions 2 and 5 allowed visual access to the CS^+^ or the CS^−^, and regions 1 and 6 were those neighboring the CS^+^ and the CS^−^, respectively. Each swimming trial was then quantitatively described by an attribute vector containing the path length (L) and the cumulative local curvature (C) for each partition (*i.e.* 1 to 6). Subsequently, the attribute vectors from all swimming trials were pooled together and fuzzy-clustered, using the first eight principal components. In other words, we typified each swimming path with respect to the center of mass of each cluster, defined by a specific constellation of behavioral attributes (Fig. S5C). In the group averaged cluster maps, their similarity to each of the eight clusters was represented in color and piled up vertically (y-axis) as a function of the training units (x-axis), for correct (left) and incorrect (right) swims (Fig. 5C). To align these results to a single reference, we subtracted the average of the eight cluster weights from the last 30 trials of the SSIM_0.04_ group from each cluster map, and then averaged the sum of the referenced values from all clusters (Fig. 5D). In these line plots, consequently, a conditioned response approached ‘optimality’ when it differed less from the average performance level reached at the end of SSIM_0.04_ (dotted line). Two-way repeated-measures ANOVAs followed by *post hoc* tests revealed that the mice learned to solve the task differently during acquisition, depending on how stimulus similarity varied over the consecutive learning trials (*P < 0.05 below Fig. 5D). In the same way, the point-to-point subtraction (−1 in blue and +1 in red) between the cluster maps of the [increasing]-[decreasing] training regimes, revealed clear group differences in the structure of the swimming paths (Fig. 5E).

At the end of training, the referenced conditioned responses (Fig. 5F–H; Wilcoxon test, *P < 0.05, **P < 0.01) and their relative differences (depicted by lower case letters, *P* < 0.05 for all pairs, one-way ANOVA tests, followed by Bonferroni's post hoc tests or Kruskal-Wallis test followed by Dunn's post hoc tests; see Methods for details) were fully consistent with our previous choice comparisons (Fig. 3E–G), yet the differences in the incorrect swims (Fig. 5F, G) indicate that important correlates of learning were stored in the incorrect choice records.

### Transitions between response variants

By careful inspection, we realized that the mice could use multiple strategies to solve the task^4^,^33^. For instance, in some trials the CS^+^ image could not be used as a reliable predictor of the platform due to the high degree of stimulus similarity. In such scenario, the mice displayed alternative solutions to the task such as randomly choosing either arm of the pool or swimming repeatedly to the same arm (*i.e.* side bias)^3^,^33^. We analyzed our training programs and found that solving the task in a side biased manner during the entire training phase would be more efficient than making random or other choices (completely biased: 537.5 ± 0.2 trials/300 training units, *n* = 1000 ‘subjects’; random choosing: 590.5 ± 0.7 trials/300 training units, *n* = 1000 ‘subjects’; ‘following last CS^+^’: 765.5 ± 0.1 trials/300 training units, *n* = 1000 ‘subjects’, one-way ANOVA, *F*_2,2996 _ = 2684.2, *P* < 0.001). However, we also noted that biased and random choosing would produce average choice values around chance level (completely biased: % correct = 50.1% ± 1.6%; random choosing: % correct = 50.1% ± 1.5%; ‘following last CS^+^’: % correct = 34.83% ± 4.8%), indicating that these two scenarios could not be differentiated by comparing correct choice distributions. These implicit differences in task efficiency derive from the fact that the position of the CS^+^ and platform (left or right) was continuously exchanged according to a Gellerman schedule^3^,^4^, which prohibits their placement in the same arm for more than three consecutive trials. This means that choosing the same arm would produce a correct choice after a maximum of three error repetitions, whereas other less efficient strategies, like random choosing or following the last position of the platform, can reach up to five error repetitions. Altogether, these numbers highlight the fact that our training regimes could lead to multiple response variants, with different swimming efficiencies arising from selective usage of discriminative information (see also Fig. S4B).

**Figure 6:**
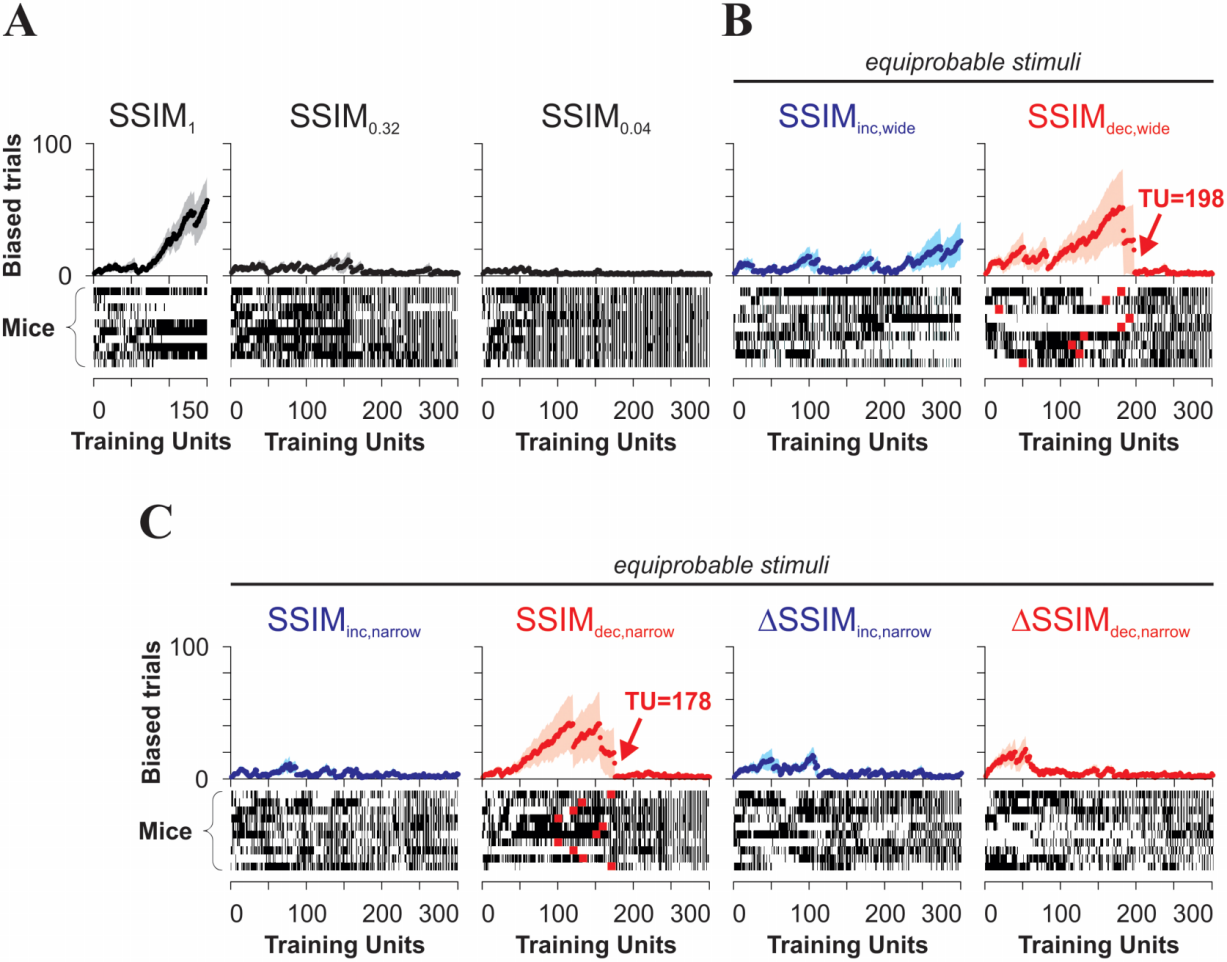
Transitions between complementary task-solving strategies during visual discrimination learning. Alternative task-solving strategies emerge depending on the extent to which the CS^+^/CS^−^ images become a reliable source of information for behavioral control. (A–C) Side-bias (group average) as a function of training for the different groups (see Methods). At the bottom of each panel, the choices from individual mice (y-axis) are shown as either black (right choices) or white (left choices) rectangles as a function of training unit (x-axis). Individual side-bias is reflected as horizontal white or black blocks of different lengths. The red squares in the SSIM_dec_ panels depict the location of the training unit at which each subject presented the biggest drop in side-bias. Note how the mice trained with a constant SSIM of 1 slowly adopted a side-bias (A).

We hypothesized that the probability of displaying CS^+^-independent (*v.gr.* biased) behavior should depend on CS^+^/CS^−^ similarity and on how it changed during training. We quantified the strength of the ‘side bias’ for each experimental group as the average number of consecutive trials in which the mice systematically swam to the same arm of the pool, either left or right, reflecting the lack of engagement in the discrimination task. Strikingly, we found that in SSIM_1_, where visual discrimination was impossible (see Fig. 2A), the mice progressively learned to become side biased (Fig. 6A), suggesting a slow increase in task efficiency (compared against random-choice behavior; see also Fig. S4B). By contrast, the mice trained on a regime with decreasing similarity showed an initial increase followed by a rapid drop in side bias, indicative of a sharp transition to CS^+^-dependent behavior (Fig. 6B, C). Side bias was much smaller in the groups trained with increasing similarity, and it was dramatically smaller in ΔSSIM_dec,narrow_ than in SSIM_dec,narrow_ (Fig. 6C). Further inspection confirmed that the rapid drops in side bias observed in the mice trained with regimes of decreasing similarity (red arrows in Fig. 6B, C) preceded the onset of successful discrimination, as detected through choice (Figs. 2B and 3A).

**Figure 7:**
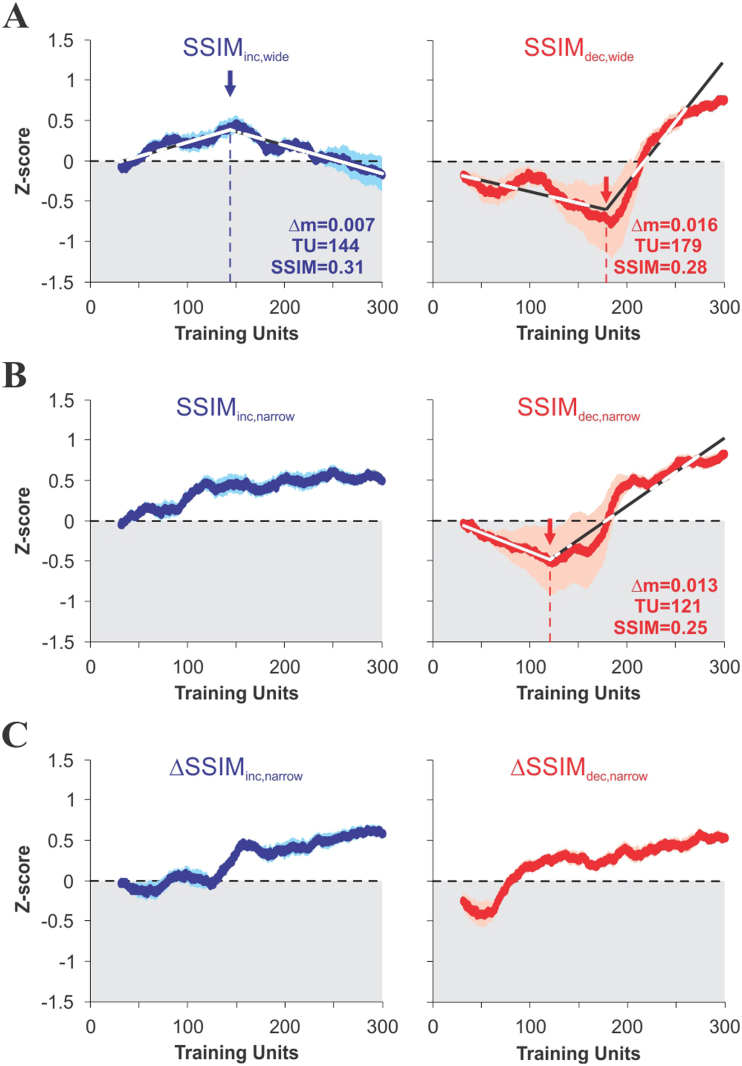
Graded transitions into visually-guided behavior. A quantitative measure ‘*z*’ is obtained by subtracting visual and non-visual factors, plotted as a function of training for groups trained with varying CS^+^ similarity (see Methods and Results). The transitions to visually-guided behavior were detected by minimizing the sum of the squared errors between the dataset and a single free-knot linear spline^34^. The strength of these transitions is proportional to the differences in the slopes around the transition points, as indicated by the arrows. Stronger transitions are observed when mice are trained with decreasing (in red) CS^+^/CS^−^ similarity, as opposed to increasing similarity (in blue; see Results for statistical details).

To estimate and compare the strength of the transitions from side-bias to CS^+^-dependent behavior, we created a quantitative measure ‘*z*’, given by the difference between two competing processes: one that accounted for correct choice *minus* another one that accounted for side bias (see Methods). We identified the time point of the transitions by minimizing the sum of the squared errors between the dataset and a single free-knot linear spline^34^ and estimated their relative strength by calculating the difference in slopes, before and after the turning points (Fig. 7). This analysis revealed that there were sharper and stronger transitions in the groups trained with decreasing rather than with increasing similarity (SSIM_inc,wide_: training unit 144, SSIM = 0.31, Δm = 0.007; SSIM_dec,wide_: training unit 179, SSIM = 0.28, Δm = 0.016; SSIM_dec,narrow_: training unit 121, SSIM = 0.25, Δm = 0.013; Fig. 7A, B), and training with dynamic gradients of inter-training unit SSIM quickly led to CS^+^-guided search (Fig. 7C). Also, the rising slope into CS^+^-guided behavior was much steeper in SSIM_dec,wide_ than in SSIM_dec,narrow _(*F*_1,296 _ = 113.1, *P* < 0.001).

### Constraint in the discrimination process

**Figure 8:**
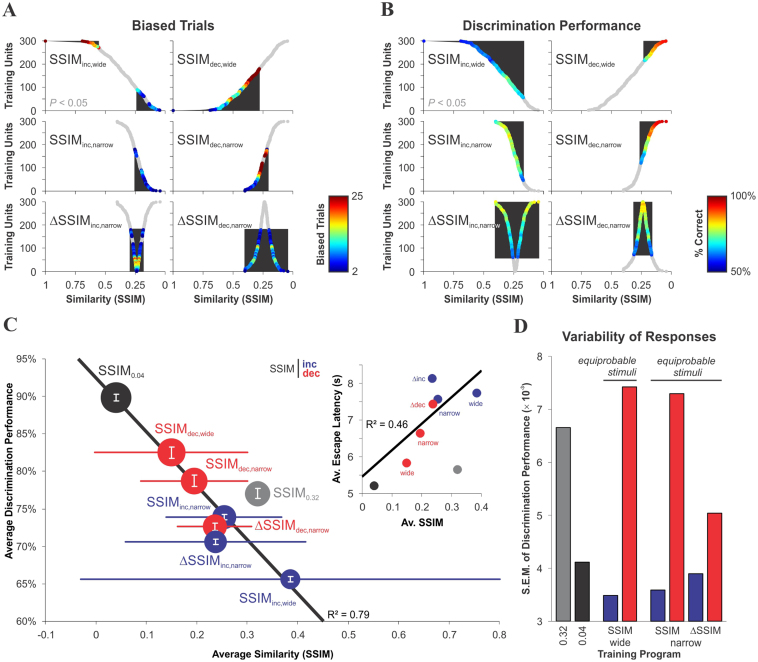
The interplay between visual discrimination and learning. Training with varying CS^+^/CS^−^ similarity exposes the flexibility of visual discrimination performance. (A–B) Complementary scatter plots for biased trials (A) and visual discrimination performance (B) as a function of training (y-axis) and CS^+^/CS^−^ similarity (x-axis). The black regions define the similarity range for biased swims (≥2; A) or successful discrimination (B). Gray dots represent unbiased choices (A) or choices undistinguishable from chance level (B, color-bars on the right; see also Methods). (C) Average discrimination performance from the complete set of discriminative trials against the average stimulus similarity during training. The vertical bars are the S.E.M. of the discrimination performance (amplified in D), while the horizontal bars represent the SSIM-range for successful discrimination (identical to shaded regions in B). Dot-size is proportional to average efficiency (see Fig. S4). The plot reveals a compromise: training with decreasing similarity leads to higher levels of discrimination performance but covering a smaller similarity range for successful discrimination, as opposed to training with increasing similarity. The upper inset shows that in discriminative trials the average escape latency increases with the degree of CS^+^/CS^−^ similarity. In this task, the mice benefit from having quick reaction times, because the platform (reward) is spatially dissociated from the regions where visualization of the stimulus occurs and, as a result, speed might be favored over accuracy. Continuous lines are weighted linear regressions. (D) Despite the lower discrimination performance, training with increasing similarity presents a more precise pattern of discrimination choices.

The compromise between discriminability and discrimination learning can be visualized when training (y-axis) is plotted with varying stimulus similarity (x-axis) against either biased trials (Fig. 8A) or successful discrimination performance (Fig. 8B; displayed in color; see also Fig S4B). The shaded regions in these figures frame the variable range of stimulus similarity observed for biased and discriminative trials. Plotting the relationship between the average discrimination performance (y-axis) against the average CS^+^/CS^−^ similarity during training (x-axis) reveals a crucial property of the discrimination process (Fig. 8C). Namely, training with decreasing similarity rendered higher average discrimination performance than training with increasing similarity, although it did so at the expense of less precision (Fig. 8D) and over a smaller similarity range (represented by the horizontal bars; Fig. 8C). Conversely, lower discrimination performance was associated with more flexibility in resolving difficult discriminative operations. This increased the range (and average similarity) for successful discrimination and average escape latencies (Fig. 8C, inset^30^,^35^, but see^31^), in agreement with predictions^36^,^37^,^38^.

## Discussion

In this study, we addressed the interplay between discrimination and learning. We adapted a two-alternative forced-choice visual discrimination task^3^,^4^ and trained freely moving mice to discriminate between a constant CS^+^ (reinforced) and a varying CS^−^ (non-reinforced) image, exchanged over consecutive trials. We used heterogeneous CS^−^ images with signals broadly distributed over different spatial frequencies, below the mouse visual acuity threshold^3^,^4^. To manipulate CS^+^/CS^−^ discriminability, we selected specific CS^−^ stimuli with different degrees of structural similarity to the CS^+^, measured using parametric descriptions derived from image quality metrics. Hence, we employed such measures to arrange the same stimuli into different configurations of variable discriminability. As expected, when CS^+^/CS^−^ similarity remained constant, learning rates and peak performance were negatively correlated to CS^+^/CS^−^ similarity^22^,^36^,^37^,^38^,^39^. However, when the mice were trained with equiprobable training stimuli covering a wide range of varying CS^+^/CS^−^ similarities, then the sign of the sustained SSIM gradients (either positive or negative) produced markedly different learning profiles. Specifically, training with negative similarity gradients was associated with faster learning, higher (albeit less precise) average choice performance and slower extinction than with positive gradients. This indicates that the animals exposed to the different, yet comparable, training conditions learned to discriminate items from the visual stream in different ways (see also^8^). Notably, the group trained with increasing similarity also reached above random choice level performance at the very end of training, when CS^+^/CS^−^ similarity reached maximum values, indicating that the stimuli still carried enough discriminative information for this specific group of mice. Because initial SSIM values were un-restrained and covered a wide range of similarity, we used data on discrimination performance from the mice trained with these stimuli to determine psychophysical thresholds for visual discrimination. Using the upper limit of these thresholds, we created a new set of hybrid CS^−^ stimuli covering a ‘narrow’ CS^+^/CS^−^ similarity range, which did not compromise CS^+^ discriminability. Training with equiprobable stimuli covering this narrower range yielded similar results as training with the wide range, although with smaller differences between groups. The two groups of mice trained with decreasing similarity (SSIM_dec,wide_ and SSIM_dec,narrow_) learned to discriminate very similar images with similar performance level (≥80%), despite the fact that discrimination was potentially compromised at the beginning of training in SSIM_dec,wide_, due to the low stimulus discriminability. It is likely that in SSIM_dec,wide_, the unsuccessful discrimination attempts that occurred at the beginning of training contributed to learning by ‘boosting’ discrimination performance as soon as CS^+^ discriminability crossed over certain threshold (see also^24^).

Present models of decision behavior rely on the idea that discriminative decisions are based on sensory evidence that is integrated over time (with some degree of noise and leakage) until the representation of one of two mutually exclusive alternatives reaches a critical level^13^,^14^,^27^,^28^,^40^,^41^,^42^. This probabilistic sampling assumption predicts that learning is expressed as a gradual improvement in behavioral performance over successive trials, superimposed on significant variability from one trial to the next. In line with this, we found that path-length, escape latencies and their variability, asymptotically decreased as learning progressed, although these measures were also highly sensitive to the different training regimes. Our quantitative analysis of conditioned response development not only confirmed the group differences arising from correct choice, but it also revealed that important learning correlates were stored in the error trials. Moreover, we also found that the mice could use multiple task-solving strategies with different efficiencies^4^,^33^. For instance, they could solve the task not only by using the CS^+^ as a relevant source of information for behavioral control (a highly efficient strategy), but they could also display side bias swimming (a strategy of medium efficiency), or simply choose randomly (a weak strategy). Using this characterization, we went one step further and found that drops in side bias, which were detected individually, preceded the onset of CS^+^-dependent behavior. Side bias was much less prevalent in the groups trained with increasing similarity, while the groups trained with decreasing similarity initially showed greater bias and subsequently, displayed sharper and stronger transitions into CS^+^-guided behavior. To our surprise, training with oscillating gradients of inter-training unit SSIM quickly led to CS^+^-guided searching and side bias was dramatically reduced in ΔSSIM_dec,narrow_ when compared to SSIM_dec,narrow_. Altogether, our characterization of the transition into CS^+^-guided behavior demonstrates that both sensory and non-sensory information influenced choice behavior^8^,^43^,^44^ (see also^13^). Such measures may become useful to detect early stages of visual discrimination learning and correlates of attention deficit disorders^45^.

The differences in learning rate and side bias between the SSIM_dec,wide_ and SSIM_inc,wide_ groups, suggest that attention signals and/or diffuse neuromodulatory systems were differentially engaged in these groups^9^,^46^,^47^. It has been reported that attention functions as a gate to ensure that visual perceptual learning occurs only in response to features to which attention is directed (task-relevant features), enhancing the stream of signals to specific brain areas^24^. Moreover, attention is frequently seen as a perceptual filter that limits access to awareness and memory^48^, and theoretical work anticipates that sensory representations are dynamic and that they interact in a non-linear manner to compete to enter into a limited-capacity attention buffer^25^,^36^,^37^,^38^. Interestingly, our results uncovered an inverse relationship between discrimination performance and discriminative resolution. This constraint is in agreement with the idea of limited computational resources to solve the discrimination task and implies that sensitivity within a similarity range cannot be improved without sacrificing performance in another^49^. An attractive proposal is that learning easy (or low precision) tasks is linked to plasticity in higher level cortical areas which generalizes across locations and feature values, whereas training in high precision tasks is more specific to the trained stimulus implicating lower representational levels (for review see^8^).

The possibility of transforming indiscriminable stimuli into discriminable perceptions accentuates the potential of learning to enhance sensory perception and support adaptive behavior^50^. One of our main results is that the discrimination threshold was determined by controlled variations in similarity during visual learning. Again, this suggests that cortical mechanisms might have been differentially engaged in our experimental groups^11^,^39^,^50^. In addition, the overall correct choice level in discriminative learning trials was negatively correlated to CS^+^/CS^−^ similarity, which indicates that the discrimination process can be approximated by a subtraction operation^19^,^28^,^49^,^51^, at least from a steady state perspective. If one adopts this view, solving the task would involve a three-step sequence of causally-linked events: i) encoding the CS^+^ and CS^−^ stimuli, ii) inferring correct choice probability based on the structural difference between both stimuli, and iii) deciding according to the sign of such difference.

Problems in estimating psychometric sensitivity have been largely ignored in the literature on the physiology of perception. In most neurophysiological experiments, animals are presented with more than two stimuli, varying their discriminability, and usually responses are analyzed after performance ‘no longer improves’^52^. However, psychophysical performance, measured in terms of the proportion of correct responses, may differ in different tasks, and even within the same task^53^. Using identical sensory information arranged in different combinations during training, we here explored and described how learning interacts with measured discriminability. Our results highlight the fact that sensory and non-sensory factors influence discrimination performance and compellingly demonstrate how mice use their visual system to perform behaviorally-relevant computations with explicit control of visual information. We described here a new training protocol with complex visual stimuli and provided the analysis tools to detect discrimination thresholds and the transition from sub-optimal conditioned response variants into discrimination-guided behavior. These methods are useful to analyze visual discrimination, perceptual learning and selective attention in mice.

## Methods

### Animals

Behaviorally naïve, wild-type C57BL/6 male mice (*n* = 88, P40–50, 21 ± 3 g at the start of experiment; Charles River, Sulzfeld, Germany) were trained in a two-alternative visual discrimination task (Fig. 1). The mice were housed individually at 22°C under a 12/12 h light/dark cycle with standard sleeping periods, 35–40% relative humidity, *ad libitum* access to food and water and a fast-track device for physical exercise (PLexx, Netherlands). The mice from different litters were randomized into 9 groups according to the different training regimes (see below). Groups of 4–6 mice were handled by a single experimenter and they were habituated to the training room 3 days before the experiments began. The experiments were performed during the light phase (between 10:00 and 16:30), over a single daily session consisting of 3 blocks of 10 ‘training units’ (see below) with 10 min breaks, 5 days a week, during 4 weeks. All experiments were carried out at the Max Planck Institute for Medical Research in accordance with the animal welfare guidelines of the Max Planck Society and were approved by the regional commission in Karlsruhe (G-171/10).

### Generation of equiluminant stimuli

300 pictures were downloaded from the internet, transformed into black and white pixels, centered on a white 22 × 22 cm square and iteratively scaled up/down until the cumulative surface areas of the black and white pixels were identical (tolerance = 0.01%). The resulting images consisted of white shapes on a black background, or *vice versa* (*i.e.* the shape was the only relevant ‘feature’). The images were of similar size, and were further standardized by using a symmetric Gaussian low-pass filter (60 pixel size, 30 pixel standard deviation; ~0.30 cycles per degree [c/d]), removing all the frequency components that spanned beyond the mouse's visual acuity of ~0.48 c/d^4^. The structural similarity index (SSIM; see below) of this set of images ranged from −0.07 to 1. A second set of images was created artificially to increase the number of stimuli with lower structural similarity (0.04 ≤ SSIM ≤ 0.39 against our chosen CS^+^). To do this, linear combinations of image pairs from the original set were re-scaled, re-filtered and selected by using nearest neighbors to random numbers from a known Gaussian distribution. As a result, the images from both sets were equiluminant (Set_1_: 85 ± 1 lux, *n* = 300; Set_2_: 84 ± 1 lux, *n* = 300; measured at a distance of 46 cm and at water level; Voltcraft MS-1500, Hirschau, Germany), making the visual discrimination task independent of brightness information (Fig. 1E, F). We confirmed that in our experimental conditions, visual discrimination was invariant to visual cues other than those displayed by the monitors^4^.

### Image quality metrics

We used image quality metrics (IQM) to compare the degree of similarity between our training stimuli. IQM refers to algorithms that measure the visual equivalence of two images^54^ representing a quantitative set of measures that predict the perceived image quality: when physical differences (*i.e.* pixels) become visible differences perceived by human observers. These are algorithms that are broadly applied for image acquisition, compression, communication, displaying, printing, enhancement, analysis and watermarking. We implemented 5 ‘statistics-oriented’ measures (*i.e.* based on signal differences): 2D-cross correlation (XCORR = ΣΣ(reference_image_1_·query_image_2_)/(σ_1_σ_2_)), mean-squared-error (MSE = (1/N)·Σ(reference_pixel_i_-query_pixel_i_)^2^), signal-to-noise ratio (SNR = 10·log_10_·((1/N)·Σ(reference_pixel_i_)^2^/MSE)), peak signal-to-noise ratio (PSNR = 10·log_10_·(255^2^/MSE)), weighted signal-to-noise ratio (WSNR^55^), and 4 ‘visual-system-oriented’ measures (*i.e.* based on structural information loss): a structural similarity index (SSIM^20^), visual information fidelity (VIF^56^), pixel-based VIF (VIFP^56^) and noise quality measure (NQM^57^).

### Visual discrimination learning

We used a well-established two-alternative, forced choice, water discrimination task (see^3^,^4^), under a ‘free response’ paradigm, whereby the mice are allowed to control the decision time autonomously. In this task, mice were trained to visually discriminate between two images displayed simultaneously on two separate monitors, and to learn that swimming towards the reinforced image (CS^+^) and reaching a transparent submerged platform (US) was rewarded with escape from water, whereas swimming towards the non-reinforced image (CS^−^) was not. Once the choice line was crossed, which limits a decision area offering visual access to both images, the subjects were considered to have made a choice. Learning was inferred by correct choice and conditioned responses. To avoid positional learning, the side of the CS^+^ and platform (left or right) was continuously changed according to a Gellerman schedule^3^,^4^. To encourage discrimination learning, we increased the cost of making mistakes, as swimming trials with incorrect choices were immediately repeated until the animal made a correct choice with a maximum of 5 errors. These sets of swims, ranging from 1 to 6, constituted a ‘training unit’, and they involved the same pair of CS^+^/CS^−^ images (Fig. 1C). While the inter-trial interval was of 10 s, the period between training units was 1–2 min (*i.e.* distributed practice) and short training sessions of 30 daily training units were used^4^. The mice remained in the platform for 30 s before being removed from the pool. During resting periods, the mice were transferred to individual chambers with a warm plate. The water temperature (21 ± 1°C) and room illumination were kept constant throughout the experiments, and the pool was wiped down daily with 70% ethanol. The experiments were conducted in silence. Animals were behaviorally naïve to the task and began the training phase displaying correct choices at chance level. Each experiment involved three phases. During phase 1 (*i.e.* ‘pre-training’, duration: 1 week; training units 1–150), the mice familiarized with the swimming task and they learned to assign a CS_0_^+^ image with a predictive value (CS^−^ was 50% gray). During phase 2 (*i.e.* ‘training’, duration: 2 weeks; training units 151–450), the mice were trained using regimes of different and varying CS^+^/CS^−^ similarity. During phase 3 (*i.e.* ‘extinction’, duration: 1 week, training units 451–600), we monitored the decay of the conditioned response at low SSIM in the absence of reinforcement (*i.e.* with the platform removed). Balanced experimental conditions were intermingled (*i.e.* increasing vs. decreasing SSIM) and all the groups (sample size ~10 mice) shared the same CS^+^_0_ during phase 1, the same CS^+^ during phase 2 and 3 (different to that of phase 1), and the same CS^−^ during phase 3. Performance at the end of pre-training reached maximum level within the two last blocks of 30 training units (one-tailed paired-tests, *t*_87_ = 1.5, *P* = 0.07), did not vary across groups (Kruskal-Wallis tests followed by Dunn's multiple comparison tests, *H*_9 _ = 12.9, *P* = 0.12) and did not correlate with the performance during the second phase (paired-t tests followed by Pearson correlation, *P* < 0.01). To estimate the amount of human error during training, we visually reanalyzed a random sample of 10% of all swimming trials (per mouse) and found that the average error rates were relatively low (mischaracterized choices: 3.04% ± 1.00%; misplaced platform: 1.52% ± 1.29%) and trainer-dependent.

### Behavioral analysis

For each pair of CS^+^/CS^−^ images, we calculated the mean probability (± S.E.M) of making a correct choice on the first presentation (% Correct), and of making 5 consecutive errors (% Error) with the same pair of images. The change of these probabilities over the training units was approximated by a Savitzky-Golay filter (span = 25 trials, degree = 1), which served as a low-parameter estimate to visually compare group data from different training regimes. Learning (also referred to as acquisition) rates were calculated as the maximum performance level divided by the number of training units required to achieve such a level after successful discrimination was detected (*i.e.* above chance level). We used a digital video camera mounted above the pool to record each swimming path throughout the entire set of experiments. For each trajectory, we analyzed continuous measures of path length (*i.e.* cumulative Euclidean distance) and the escape latency (*i.e.* the time of release from the chute until completion of the task). These measurements are strongly task-dependent^30^,^31^. To examine the data from the extinction phase of the experiment, we calculated a ‘retention index’, given by the accumulated distance travelled in the CS^+^-arm divided by the total path length (Fig. 4). A local measure of path curvature (in °/pixel) for each discrete position X within the path was computed as the angle between AX and BX divided by the path length of the segment contained in R, where A and B are the extreme points that occur in a circle of radius R = 15 pixels = 6.66 cm around X. We discovered that multiple path structures with variable curvature occur in conditions that range from apparent random to ‘optimal’ CS^+^-guided search. To analyze and categorize these conditioned strategies, pattern vectors from all swimming trials were split into ‘regions of interest’ in the pool (Fig. 5 and Fig. S5). Next, they were joined together and clustered with a fuzzy C-means algorithm, using the first 8 principal components. We quantified the strength of the side-bias as the number of consecutive swimming trials that the mouse swam towards the same arm of the pool (either right or left), showing invariance to the CS^+^ position. A ‘z’ score was computed as the sliding average of [choice-50%] – k*[Bias], over blocks of 30 training units for each mouse, with an arbitrary k = 1/50 for all groups (the turning points of ‘z’ are independent of k; Fig. 7) and combined linear regressions were fit by minimizing the sum of the squared errors between the dataset and a single free-knot linear spline^34^. Swimming efficiency was calculated as E = 1−(([X]−1)/(X_random_−1)), where X is the group average number of swimming trials per training unit and X_random_ corresponds to the average number of swimming trials required to complete each training unit by making random choices (R_1000_: binomial distribution, *n* = 1000 ‘subjects’; see Fig. S4). Analysis, stimulation and video-tracking algorithms were designed and written by M.T., while the interface to collect attributes, select and display swimming traces was designed by M.T. and written by P.J. in MATLAB 7.8 (MathWorks, Inc.; Natick, USA).

### Statistical analysis

Choice, conditioned responses and comparisons between two-groups were assessed with one-sample t tests. Multiple group comparisons were performed with one-way ANOVA tests, and maximum performance levels and the probability of adopting a side-bias (≥2 consecutive trials) with repeated measures ANOVA tests (consecutive blocks of 15 or 30 training units), all followed by Bonferroni's *post hoc* tests. We switched to nonparametric tests (*i.e.* Wilcoxon Signed Rank test; Kruskal-Wallis test followed by Dunn's post hoc tests) whenever the assumptions required to use the parametric versions were not met. All results are shown as the averages ± S.E.M and significance was set at *P < 0.05 or **P < 0.001.

## Author Contributions

M.T. and R.D.M. conceived and designed research; M.T., T.O., P.J. and S-B.L. performed experiments; G.K. provided material and space for the experiments; M.T. and G.K. supervised the project; M.T. designed stimuli and analysis algorithms; M.T. and R.D.M. analyzed data; M.T. drafted the manuscript and made figures; M.T. and R.D.M. wrote the manuscript; all authors revised and approved the final version of the manuscript.

## Supplementary Material

Supplementary Information Supplementary Information
