# Supplementary material for: Controlled variations in stimulus similarity during learning determine visual discrimination capacity in freely moving mice
Source: Sci Rep. 2013 Jan 10;3:1048. doi: 10.1038/srep01048 (PMC3541512; doi:10.1038/srep01048)
Supplement: Supplementary Information [file srep01048-s1.pdf]

Controlled variations in stimulus similarity during learning determine visual discrimination capacity in freely moving mice

Mario Treviño[1*], Tatiana Oviedo[1], Patrick Jendritza[1], Shi-Bin Li[1], Georg Köhr[1] and Rodrigo J. De Marco[2*]

[1]Department of Molecular Neurobiology and [2]Developmental Genetics of Nervous System
Max-Planck-Institute for Medical Research, Heidelberg, Germany

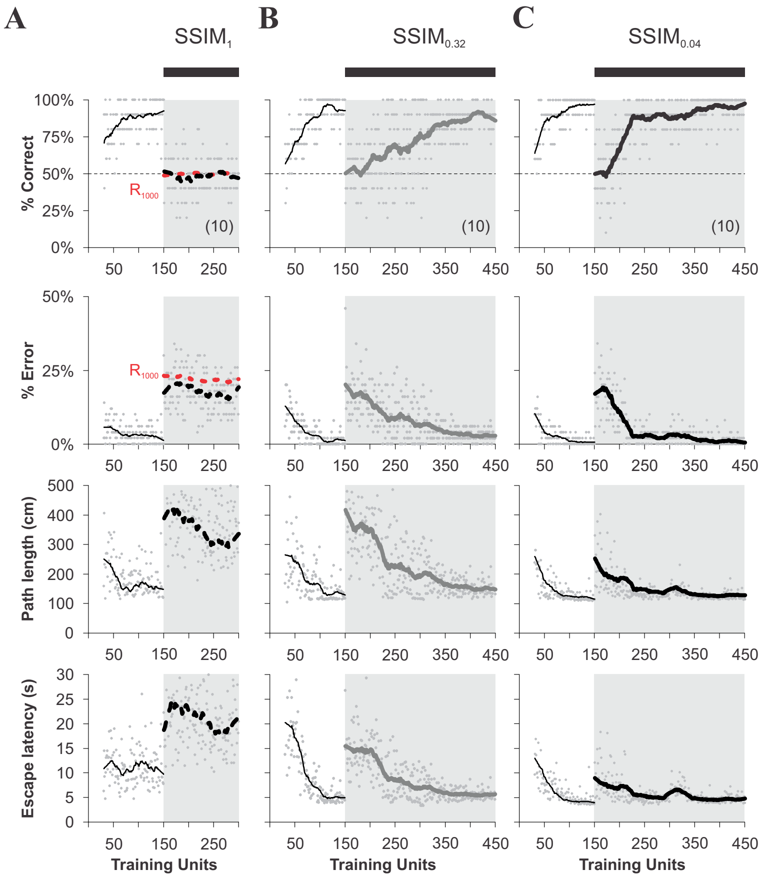

Figure S1. Discrimination learning with constant CS⁺/CS⁻ similarity. The mice familiarized with the swimming pool and the general requirements of the task during an initial week of 'pre-training' (phase 1: trials 1-150), and learned that a highly discriminative CS^+ image (CS^- was a 50% gray screen) predicted the location of the platform. During the second and third week of the experiment (phase 2: trials 151-450), the mice learned to make visual discriminations over the course of acquisition while being exposed to fixed CS^+/CS^- similarity (SSIM) levels of either SSIM = 1 (A), SSIM = 0.32 (B) or SSIM = 0.04 (C). Random choice behavior depicted by the red dotted line (R_{1000}: binomial distribution, average of n = 1000 'subjects'). Learning is inferred and reflected by the group average of different indices as a function of the training; from top to bottom: correct choice (%), number of errors (%), path length (cm) and escape latency (s) (gray dots). The average learning curves (gray and black continuous lines) are approximated by a Savitzky-Golay filter (see Methods). As learning progressed, both the length of the swimming path to reach the platform ('path length') and the mean time from the beginning to the end of the trial ('escape latency') decreased asymptotically. Note how correct choices remained at chance level with SSIM = 1, although learning rates increased with lower SSIM values. Number of mice in parentheses.

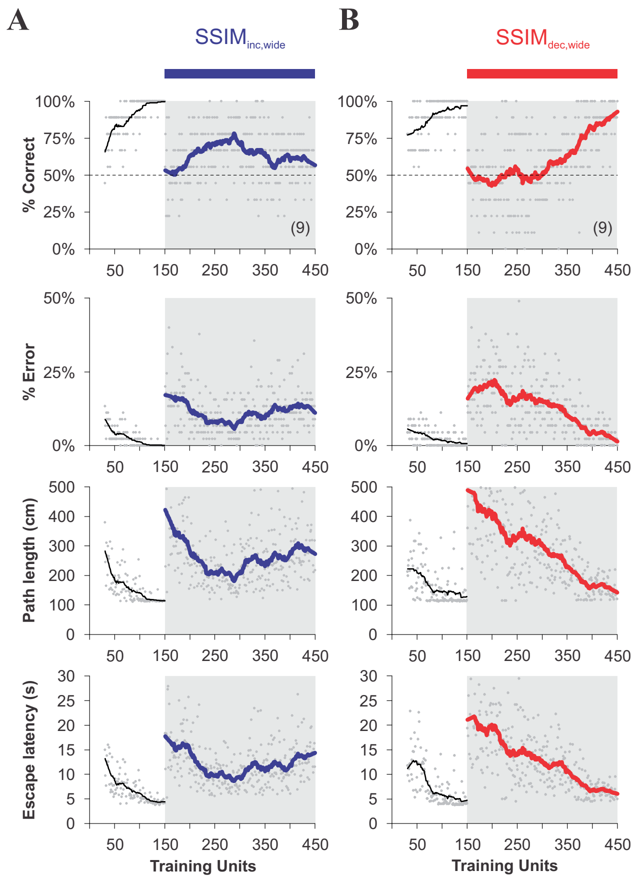

Figure S2. Discrimination learning with varying CS$^+$/CS$^-$ similarity ('wide' range). The results are displayed using the same format as in **Fig S1**. Acquisition curves for mice trained either with increasing (A, blue lines) or decreasing (B, red lines) structural similarity (SSIM) between CS$^+$ and CS$^-$ stimuli during phase 2 (trials 151-450). CS$^+$/CS$^-$ similarity ranged from -0.07 to 1 ('wide' range of SSIM). Note that the two training programs consist on the same set of equiprobable CS$^-$ stimuli, linearly sorted into increasing or decreasing SSIM values (*i.e.* sustained positive or negative gradients).

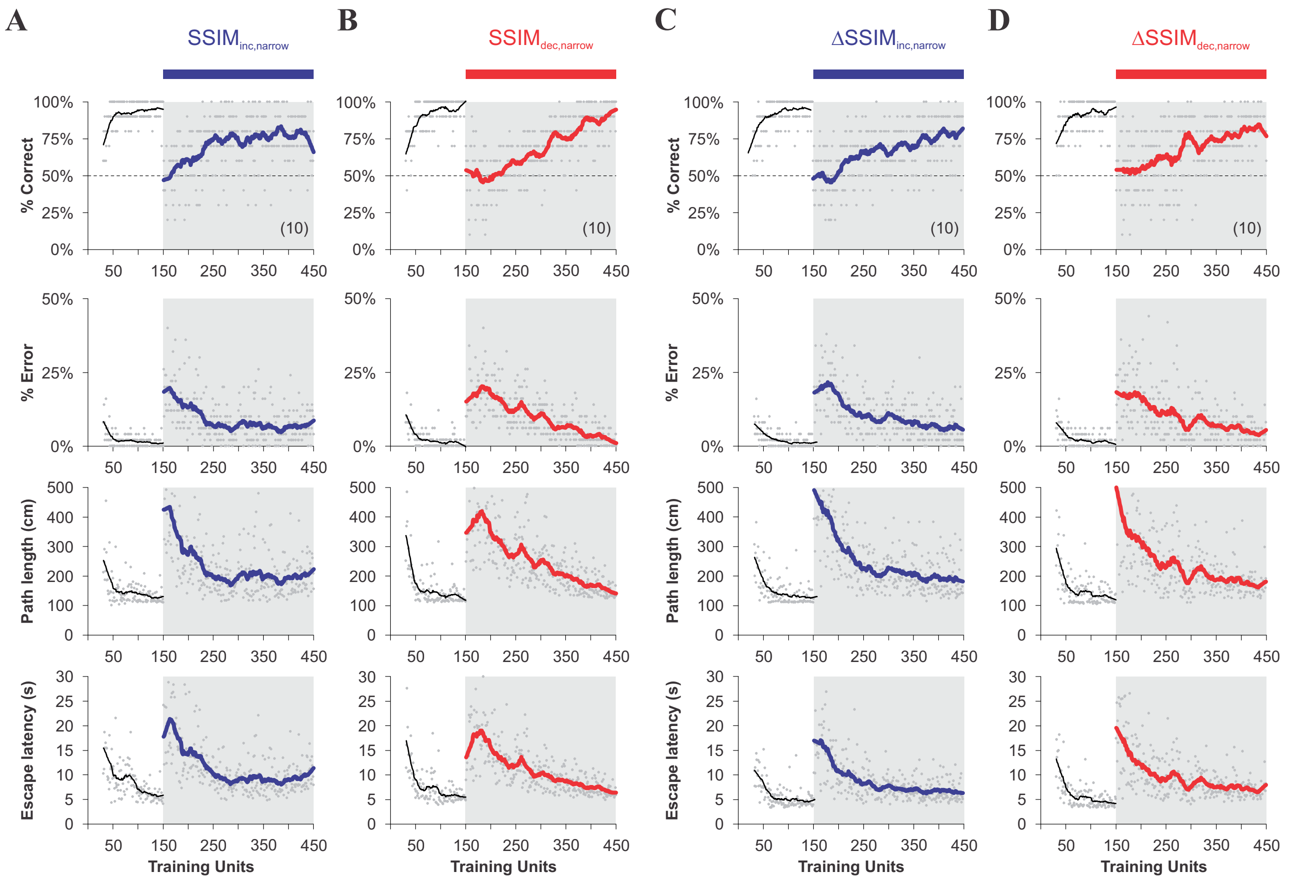

Figure S3. Discrimination learning with varying CS$^+$/CS$^-$ similarity ('narrow' range). The results are displayed using the same format as in **Fig S1**. CS$^+$/CS$^-$ similarity ranged from 0.04 to 0.39 ('narrow' range). During the second and third weeks of the experiment (phase 2: trials 151-450), the CS$^+$ and CS$^-$ images were arranged with increasing (A, blue lines) or decreasing (B, red lines) similarity, or with increasing (C, blue lines) or decreasing (D, red lines) oscillating gradients of inter-training unit similarity (ΔSSIM). The four training programs are based on the same set of equiprobable CS$^-$ stimuli, sorted into increasing or decreasing SSIM and increasing or decreasing ΔSSIM values.

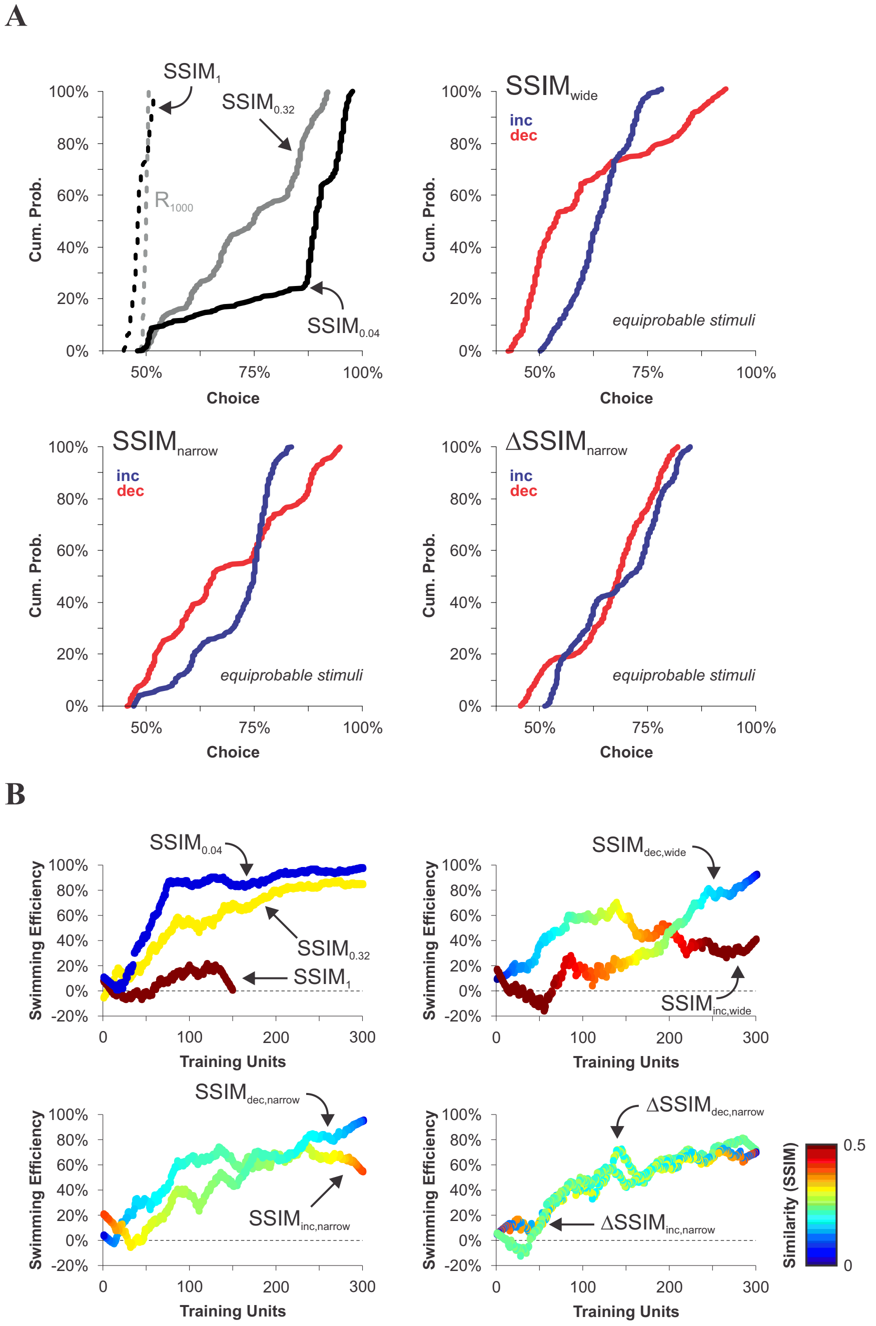

Figure S4. The interplay between learning and visual discrimination determines the average probability to discriminate. (A) Cumulative probability plots for discrimination choices obtained with the different training regimes (sorted data from **Figs 2** and **3**). The overall efficiency of the learning process is reflected as a rapid and larger increase in the net proportion of correct choices (x-axis). Note how discrimination performance depends on the training regime, as well as on the sign of the SSIM gradients used during training. Two-sample Kolmogorov-Smirnov tests reveal significant differences between the distributions in each of the four panels ($P < 0.001$ for all comparisons), and also indicate that mice trained with SSIM = 1 (black dotted line) do not choose randomly (gray dotted line, R_{1000}: binomial distribution, $n = 1000$ 'subjects'; $P < 0.001$). (B) Swimming efficiency (y-axis) as a function of training (x-axis) and structural similarity (in color; see Methods). Note the apparent increase in efficiency of mice trained with SSIM = 1, as they learned to employ a side-bias (**Fig 6A**), and how, identical SSIM values produced different swimming efficiencies depending on the training regime (Color-bar on the right).

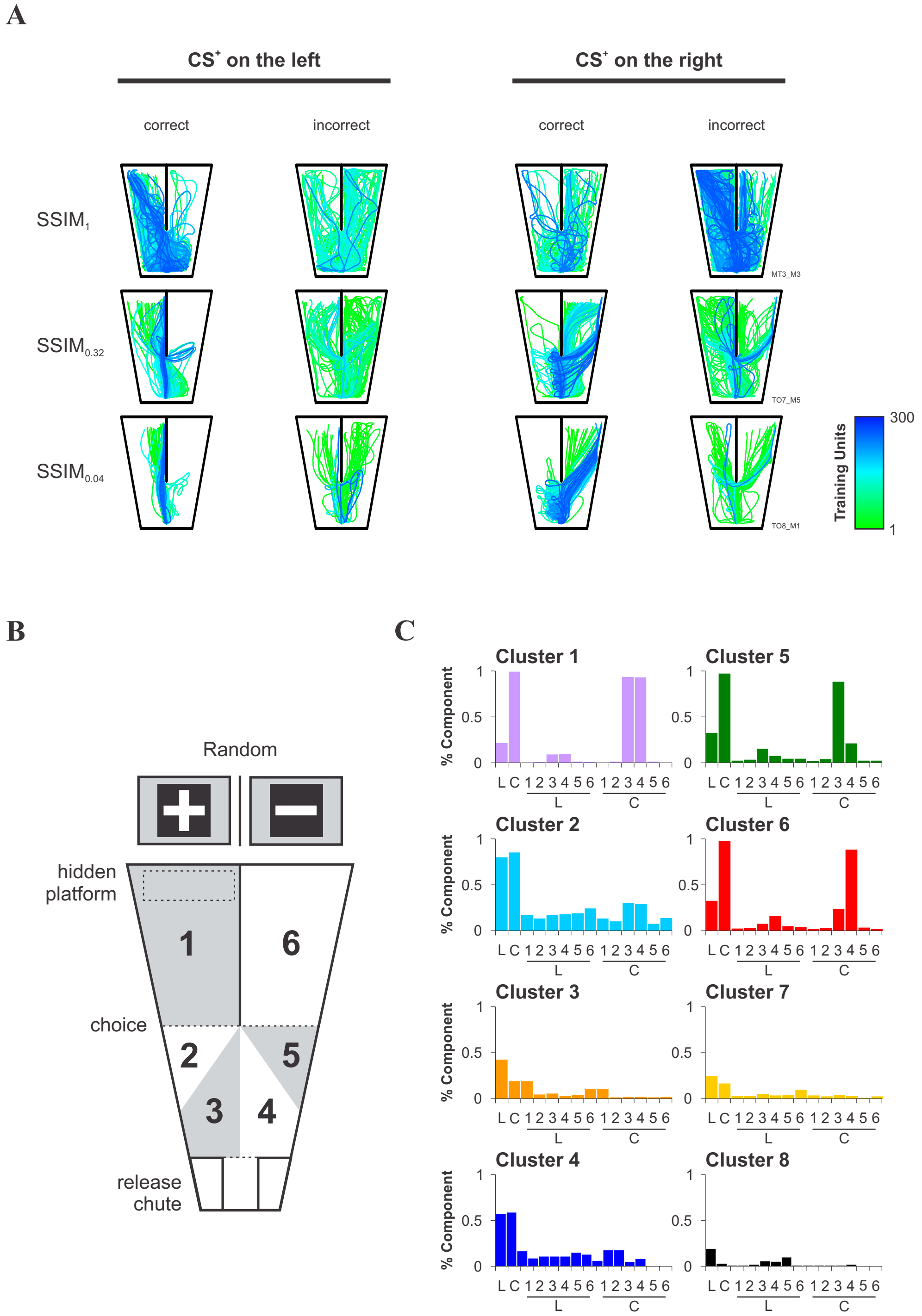

Figure S5. Cluster analysis of the swimming paths. (A) Swimming patterns from three sample mice (rows) trained with SSIM = 1 (top), SSIM = 0.32 (middle) and SSIM = 0.04 (bottom). The traces are segregated into correct and incorrect choices, when CS^+ is on the left (left panels) or on the right (right panels) arm of the maze, and are color coded as a function of training (color-bar on the right). (B) To refine our behavioral analysis, we divided the pool into six regions of interest (1 to 6) relative to the position of the CS^+ (see also **Fig 5** and Results) and each swimming trial was described by an attribute vector: [L_{total}, C_{total}, L_1, L_2, L_3, L_4, L_5, L_6, C_1, C_2, C_3, C_4, C_5, C_6], where L stands for path length and C for local curvature (*i.e.* attribute vectors contain spatial but no temporal information; see Methods). (C) The attribute vectors from all of the swimming trials (n = 60954) were pooled together and clustered using the first 8 principal components (see Methods). Bar plots depict the center of mass of each cluster, which corresponds to a specific constellation of behavioral attributes.
